# Self-referring patients at the emergency department: appropriateness of ED use and motives for self-referral

**DOI:** 10.1186/s12245-014-0028-1

**Published:** 2014-07-16

**Authors:** M Christien van der Linden, Robert Lindeboom, Naomi van der Linden, Crispijn L van den Brand, Rianne C Lam, Cees Lucas, Rob de Haan, J Carel Goslings

**Affiliations:** 1 Accident and Emergency Department, Medical Centre Haaglanden, The Hague 2501 CK, The Netherlands; 2 Division of Clinical Methods and Public Health, Master Evidence Based Practice, Academic Medical Centre, University of Amsterdam, Amsterdam 1100 DD, The Netherlands; 3 Institute for Medical Technology Assessment, Erasmus University Rotterdam, P.O. Box 1738, Rotterdam 3000 DR, The Netherlands; 4 Clinical Research Unit, Academic Medical Centre, University of Amsterdam, J1b-118, Amsterdam 1100 DD, The Netherlands; 5 Trauma Unit, Department of Surgery, Academic Medical Centre, University of Amsterdam, Amsterdam 1100 DD, The Netherlands

**Keywords:** Emergency care, Emergency department, Self-referrals, Primary care

## Abstract

**Background:**

Nearly all Dutch citizens have a general practitioner (GP), acting as a gatekeeper to secondary care. Some patients bypass the GP and present to the emergency department (ED). To make best use of existing emergency care, Dutch health policy makers and insurance companies have proposed the integration of EDs and GP cooperatives (GPCs) into one facility. In this study, we examined ED use and assessed the characteristics of self-referrals and non-self-referrals, their need for hospital emergency care and self-referrals' motives for presenting at the ED.

**Methods:**

A descriptive cohort study was conducted in a Dutch level 1 trauma centre. Differences in patient characteristics, time of presentation and need for hospital emergency care were analysed using *χ*^2^ tests and *t* tests. A patient was considered to need hospital emergency care when he/she was admitted to the hospital, had an extremity fracture and/or when diagnostic tests were performed. Main determinants of self-referral were identified via logistic regression.

**Results:**

Of the 5,003 consecutive ED patients registering within the 5-week study period, 3,028 (60.5%) were self-referrals. Thirty-nine percent of the self-referrals had urgent acuity levels, as opposed to 65% of the non-self-referrals. Self-referrals more often suffered from injuries (49 vs. 20%). One third of the self-referrals presented during office hours. Of all self-referrals, 51% needed hospital emergency care. Younger age; non-urgent acuity level; chest pain, ear, nose or throat problems; and injuries were independent predictors for self-referral. Most cited motives for self-referring were ‘accessibility and convenience’ and perceived ‘medical necessity’.

**Conclusions:**

A substantial part of the self-referrals needed hospital emergency care. The 49% self-referrals who were eligible for GP care presented during out-of-hours as well as during office hours. This calls for an integrative approach to this health care problem.

## Background

Nearly all Dutch citizens have a general practitioner (GP), acting as a gatekeeper to secondary care. Some patients bypass the GP and present to the emergency department (ED). If the patient bypasses the GP, there are no direct financial repercussions for the GP or patient. However, all Dutch residents have a mandatory own risk or excess of at least 360 euros per year for their health insurance. For GP care, the excess does not apply. If a patient goes to the hospital, this visit may result in the patient paying the costs up to 360 euros, unless the patient has already paid the excess that year for other treatments. Approximately one third of the ED visitors in the Netherlands are self-referred, while in large inner-city hospitals, up to 70% of the ED visitors present at the ED on their own initiative [[1],[2]]. Some of these self-referrals can be treated by a GP [[3]], which would decrease the workload on a crowded ED. To make best use of existing emergency care, Dutch health policy makers and insurance companies have proposed the integration of EDs and GP cooperatives (GPCs) into one facility. Most of these integrated settings are out-of-hours centres, operating from 5.00 p.m. to 8.00 a.m. on weekdays and 24 h a day during the weekends. During office hours, patients ideally have to attend to their own GP first.

Most studies focus on self-referrals presenting out-of-hours. However, EDs are also confronted with self-referrals during office hours. Knowing self-referrals' characteristics, their chief complaints, time of presentation and motives to present to the ED may help policy makers in making decisions on how to organize delivery of primary and emergency care in inner-city EDs.

The objectives of this study were therefore to examine the appropriateness of ED use and to answer the following questions: (1) Are there differences in patient characteristics and need for hospital emergency care between self-referrals and non-self-referrals? (2) Are there differences in patient characteristics and need for hospital emergency care between self-referrals presenting to the ED during office hours and during out-of-office hours? (3) Why do self-referrals seek hospital emergency care?

## Methods

### Research design and setting

A cross-sectional observational study was conducted between 1 November 2010 and 6 December 2010 in an inner-city, level 1 trauma centre, The Hague, the Netherlands with an annual census of approximately 52,000 ED patient attendances. In the study setting, there was no GPC at the time of this study.

### Procedure

Patients' age, sex and mode of arrival were recorded by the ED registration desk. The patients were categorized by the desk clerk in patients who were referred by their GP, patients who arrived by ambulance, patients who were referred by another hospital or by a medical specialist and patients who presented to the ED on their own initiative. The latter were considered self-referrals, while the others were considered non-self-referrals. After registration, triage nurses assigned a level of acuity [[4]]. Acuity levels ranged from 1 to 5, which were dichotomized into ‘urgent’ (levels 1 to 3; life-threatening, very urgent or urgent) and ‘non-urgent’ (levels 4 and 5; standard or non-urgent) for the analysis.

We assessed the relation between acuity level and the need for hospital emergency care. A patient was considered to need hospital emergency care if he/she fulfilled one or more of the following criteria: having an extremity fracture needing plaster, admitted to the hospital and when certain diagnostic tests were performed. These diagnostic tests were seven procedures not commonly performed during GP care: blood analysis, X-ray, electrocardiogram (EKG), computerized tomography (CT) scan, magnetic resonance imaging (MRI), ultrasound and lumbar puncture. For patients arriving with an extremity problem, the records were reviewed retrospectively to assess the presence of a fracture. The criteria of ‘needing hospital care’ were based on consensus of the authors (MCL, CLB, RCL).

Additional information was collected from the patient records: day and time of presentation, chief complaint, presenting with an injury and follow-up. We defined hours from 8.00 a.m. to 5.00 p.m. during weekdays as office hours. Chief complaints were based on the triage flow charts chosen by the triage nurse. Follow-up care was the discharge code as registered in the patient record. Patients referred to the children's hospital were considered as discharged from our ED.

In the weekly ED newsletter, ED nurses were asked to contribute to the study, and 12 nurses (27% of the nursing staff) agreed. After triage, these ED nurses asked consecutive self-referrals why they had chosen to come to the ED instead of going to their own GP (during office hours) or to a stand-in GP (during out-of-hours). The ED nurses were instructed to interview all self-referrals during their shift and record the exact answer of the respondents. They were aided in doing so systematically through a mandatory field in the electronic nursing records of the self-referrals. In case of a minor, the parent (or caretaker) was interviewed.

Based on previous studies [[5],[6]], patients' motives to visit the ED instead of the GP were categorized into seven categories: accessibility and convenience, perception of need, not thought about GP, not having a regular GP, familiarity, dissatisfaction with GP and referral by non-professionals. Categorization was performed by two researchers (MCL, RCL) working independently of each other, reviewing the patient records and blinded to the other researcher's opinion. If no agreement between the two researchers was noted in the assigned categorization, the case was reviewed by a third researcher (NL) who was blinded to the opinion of the other two researchers. The category on which two of the three researchers agreed was recorded for analysis. Two categories were added during the categorization: ‘no reason’ (if the nurse was not able to obtain an answer) and ‘language barriers’ (if the nurse could not understand the patients' answer).

The study was registered and approved by the regional medical research ethics committee (METC ZWH) under number 2011-011. Informed consent of individual patients was waived by the local institutional review board. The patient dataset contained no individual identifiers to maintain anonymity of subjects.

### Statistical analysis

Patient characteristics were summarized using descriptive statistics. Differences between self-referrals and non-self-referrals and between self-referrals presenting during office hours and out-of-hours, regarding age, sex, acuity level (urgent or non-urgent), chief complaint, type of health problem (injury or no injury), diagnostic tests performed, follow-up care, having an extremity fracture and the need for hospital emergency care, were analysed using *χ*^2^ tests (categorical variables) and *t* tests (continuous variables). We assessed the relation between the need of hospital emergency care and acuity level using a *χ*^2^ test.

Independent patient characteristics, in terms of age, sex, acuity level, chief complaint and injury, predicting self-referral were identified via multivariate logistic regression using a backward elimination strategy on all patient characteristics with *p* > 0.05 as elimination criterion. Predictive Analytics Software (PASW, version 18) was used for the quantitative analyses. Effect sizes were expressed in odds ratios (ORs) with their 95% confidence limits. We used qualitative content analysis to summarize the motives for self-referral [[7]]. In qualitative content analysis, language is examined intensely for the purpose of classifying large amounts of text data into an efficient number of categories [[8],[9]].

In view of the descriptive nature of this study, we did not adjust for multiple comparisons [[10]].

## Results

### Number of patients and their time of presentation

During the 5-week study period, there were 5,003 new ED patient attendances: 3,028 (61%) were self-referrals and 1,975 (39%) were not. Of these 1,975 non-self-referrals, 597 patients (30%) were referred by the GP, 618 patients (31%) were brought in by the ambulance service and 760 patients (39%) were referred by another hospital or by a medical specialist. Of the self-referrals (*N* = 3,028), 33% (*n* = 990) presented during office hours (Table 1).

**Table 1:** Patient characteristics

	**Self-referrals (*n*** **= 3,028)**	**Non-self-referrals (*n*** **= 1,975)**
Age [mean (SD)] (years)	32.3 (18.6)	48.5 (22.1)
Age categories [*n* (%)] (years)		
0 to 15	543 (17.9)	107 (5.4)
16 to 35	1,310 (43.3)	512 (25.9)
36 to 55	783 (25.9)	590 (29.9)
56 to 75	343 (11.3)	501 (25.4)
>75	49 (1.6)	265 (13.4)
Sex, male [*n* (%)]	1,636 (54.0)	951 (48.2)
Urgent acuity level^a^ [*n* (%)]	1,174 (38.8)	1,281 (64.8)
No triage [*n* (%)]	151 (5.0)	123 (6.2)
Chief complaint [*n* (%)]		
Limb problems	898 (29.7)	266 (13.5)
Wounds and local infections and abscesses	396 (13.1)	153 (7.7)
Eye/ear/nose problems and sore throat	216 (7.1)	33 (1.7)
Headache and head injury	119 (3.9)	97 (4.9)
Shortness of breath	85 (2.8)	141 (7.1)
Abdominal pain	302 (10.0)	262 (13.3)
Chest pain	203 (6.7)	191 (9.7)
Patient feeling unwell	102 (3.4)	191 (9.7)
Psychiatric problem	21 (0.7)	54 (2.7)
Other	686 (22.7)	587 (29.7)
Injury [*n* (%)]	1,476 (48.7)	386 (19.5)
Time of registration during office hours [*n* (%)]	990 (32.7)	1,073 (54.3)
Diagnostic tests performed^b^ [*n* (%)]	1,461 (48.2)	1,522 (77.1)
Follow-up care [*n* (%)]		
Discharge without follow-up appointment	1,363 (45.0)	396 (20.1)
Hospital admission	207 (6.8)	667 (33.8)
Discharge, appointment with specialist care	822 (27.1)	704 (35.6)
Left the ED without being seen by a professional	37 (1.2)	24 (1.2)
Referred to children's hospital	49 (1.6)	4 (0.2)
Discharge, appointment with GP	550 (18.2)	180 (9.1)
Suffering from an extremity fracture	336 (11.1)	204 (10.3)
Needing hospital emergency care^c^	1,539 (50.8)	1,597 (80.9)

SD, standard deviation. ^a^Urgent, very urgent or immediate. ^b^Includes blood analysis, EKG, X-rays, CT-scan, MRI, ultrasound and lumbar puncture. ^c^Hospital admission and/or suffering from an extremity fracture and/or diagnostic tests performed. All differences in patient characteristics in relation to type of referral were significant at the *p* < 0.01 level, except for ‘no triage’ (*p* = 0.06), ‘left the ED without being seen (*p* = 0.98)’ and ‘suffering from an extremity fracture’ (*p* = 0.39).

### Self-referrals versus non-self-referrals

The results described are also presented in Table 1. Self-referrals were younger (32 vs. 49 years), more often male (54% vs. 48%) and less urgent (39% vs. 65% urgent). Self-referrals also differed in the type of chief complaint; they more often presented with limb problems (30% vs. 14%), wounds (13% vs. 8%) and eye, ear, and nose problems or a sore throat (7% vs. 2%), and were more often injured (49% vs. 20%). Furthermore, they needed diagnostic tests less often (48% vs. 77%) and were admitted less often than non-self-referrals (7% vs. 34%). All differences were significant at *p* < 0.01 except for the proportion of patients with no triage (*p* = 0.06), patients leaving the ED without being seen (*p* = 0.98) and the proportion with an extremity fracture (*p* = 0.39).

Multivariate logistic regression with age, sex, acuity level, chief complaint and injury as reason for presentation as explanatory variables in the model showed that younger age (OR 0.97); being non-urgent (OR 0.49); presenting with chest pain (OR 1.79); presenting an eye, ear, nose or throat problem (OR 4.73); and having an injury (OR 2.91) were independent predictors for self-referral (Table 2).

**Table 2:** Independent factors related to self-referral to the ED

	***B*** **(SE)**	**Odds ratio (95% CI)**	***p*** **value**
Age	−0.034 (0.002)	0.967 (0.964 to 0.970)	<0.001
Urgent acuity level^a^	−0.724 (0.072)	0.485 (0.421 to 0.558)	<0.001
Injury	1.069 (0.077)	2.912 (2.504 to 3.387)	<0.001
Chief complaint			
Eye/ear/nose problems and sore throat	1.553 (0.203)	4.725 (3.175 to 7.032)	<0.001
Chest pain	0.582 (0.118)	1.789 (1.420 to 2.255)	<0.001

*B*, the coefficient for the constant; SE, standard error around the coefficient for the constant; CI, confidence interval. ^a^Urgent, very urgent or immediate.

### Self-referrals presenting during office hours versus out-of-office hours

Self-referrals presenting during office hours (*n* = 990) were not notably different from self-referrals presenting during out-of-hours (*n* = 2,038), except that they were more likely to be injured (59.9% during office hours vs. 43.3% during out-of-hours, *p* < 0.01) and to suffer from a limb problem (36.9% during office hours vs. 26.2% during out-of-hours, *p* < 0.01).

Of the self-referrals, 51% actually needed hospital emergency care. During office hours, 55% of the self-referrals needed hospital emergency care; during out-of-office hours, this was 49%. The need for hospital emergency care was not limited to patients with urgent complaints: 47% of the self-referrals needing hospital emergency care had non-urgent complaints. For example, patients diagnosed with a fracture of the finger (needing X-ray and cast) or an Achilles tendon rupture (needing surgical repair) may be triaged as non-urgent but need hospital emergency care.

### Patients' motives to visit the ED instead of the GP

During their shifts, the 12 ED nurses asked 1,751 self-referrals (58% of the total group) to answer the question why they had chosen to bypass the GP and visit the ED. Of these 1,751 self-referrals, 295 (16.8%) were minors accompanied by a parent or caretaker.

Interviewed self-referrals were not different from the self-referrals not interviewed, with respect to age, sex and follow-up. However, interviewees less often presented with an injury (45% vs. 53%, *p* < 0.001) and were less often registered during office hours (30% vs. 35%, *p* = 0.001).

**Figure 1:**
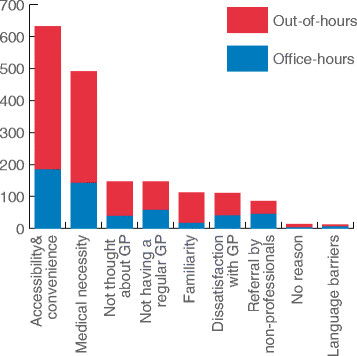
Categories of motives for choosing the ED instead of the GP.

Some patients had two different reasons for presenting to the ED instead of to their GP, resulting in 1,842 answers given by 1,751 patients. The results are shown in Figure 1. The theme that was mentioned by the respondents most often was ‘accessibility and convenience’ (632 times, 34%). Examples of answers given by participants that were placed in this category were ‘easy because the ED is always open’ and ‘not having to make an appointment’ , ‘inability to get through to the physician by telephone or get a timely appointment’ , ‘close to home’ , ‘quick service’ and ‘more flexible openings hours’. Another reason frequently mentioned was ‘perceived medical necessity’ (492 times, 27%): the perceived severity or acuity of the problem (‘too sick to go elsewhere’ , ‘emergent condition’) or the expectation that an X-ray was necessary.

‘Accessibility and convenience’ and ‘familiarity’ were slightly more often cited as motives during out-of-hours than during office hours (36.8% vs. 34.8%, *p* < 0.001 and 7.9% vs. 3.0%, *p <* 0.001, respectively). During office hours, more patients were referred to the ED by family, school, colleagues or friends (8.5% vs. 3.3%, *p <* 0.001) or stated that they had no regular GP (10.7% vs. 7.3%, *p* = 0.018).

## Discussion

In this inner-city hospital, 60% of the ED patients were self-referrals bypassing their GP because of the EDs' easy access and because they believed hospital emergency care was necessary for their complaint. They presented during out-of-hours as well as during office hours. A part of the self-referrals had medical problems eligible for GP care, and a part of them had medical problems needing hospital emergency care. Multivariate analysis indicated that the ‘typical’ self-referral was relatively young, with a non-urgent acuity level, presenting with chest pain, eye, ear, and nose or throat problems or having an injury.

Compared to previously published data varying from 3% to 76% [[1],[11],[12]], our self-referral rate was high, probably explainable by the location of the inner-city hospital. It has been shown that patients living in highly urbanized areas more commonly bypass their GPs before attending the ED [[13]].

In comparison with other studies, we had a lower percentage of self-referrals presenting with an injury [[14],[15]], and our self-referrals more often had urgent medical problems [[14]]. Although self-referrals are believed to present with problems that should be treated by their GP and cause ‘inappropriate’ attendance [[16]], both findings indicate that this is not true for all self-referrals. ‘Inappropriate’ attendance in the ED has been the subject of many studies [[5],[17]-[22]]. According to the literature, between 20% and 80% of ED visits are ‘inappropriate’ [[22],[23]]. The variability in these numbers can be explained by the variable definitions used to determine ‘inappropriateness’. The definitions of ‘emergency’ , ‘urgency’ and ‘needing hospital emergency care’ are widely debated, and medical professionals and patients differ in what they consider an ‘emergency’ and ‘appropriate visit’ [[21],[22],[24],[25]]. Given the proportion of self-referred patients in our study who did require hospital emergency care - 51% - it seems that many patients are quite capable of assessing their own need for hospital emergency care.

While the medical problems of the self-referrals in this inner-city hospital differ from those in other studies, their motives for seeking hospital emergency care are quite similar [[5],[6],[18],[26]-[29]]. ‘Accessibility and convenience’ was a major theme in our patients' decisions to bypass the GP, which could indicate a perceived and/or actual block to GP care access in the minds of the patients, even during office hours when their own GP is available.

Still, many non-urgent self-referrals and a part of the urgent self-referrals were eligible for GP care, during office hours as well as during out-of-hours. In the light of the proposed integration of EDs and GPCs into one centre, identifying patients eligible for GP care is a major challenge. A patient can be non-urgent but need complex care and a patient can be high urgent but low complexity [[30]]. For example, many GPs would consider a child with fever, triaged as urgent, eligible for GP care, while they would refer a patient with a fracture of the finger, triaged as non-urgent, to the ED for an X-ray and treatment. While an integrated ED and GPC might facilitate efficient referral between the GP and the ED, concerns have been voiced that time- and money-consuming double contacts will occur for a part of the self-referrals. Instead of diverting all self-referrals from the ED to the GP, regardless of their medical complaint or acuity level, it would be more cost-effective and patient-centred to identify self-referrals in need of hospital emergency care by triage. Ensuring that patients are treated in the appropriate setting would prevent part of the double contacts. According to van der Straten et al. [[31]], the Manchester Triage System can be used to identify non-urgent and some more urgent patients who can be treated by the GP, although triaging non-urgent patients with extremity problems to either the GP or the ED needs further elaboration.

With the high percentage of self-referrals with urgent medical complaints and the rates of self-referrals presenting during office hours, other models of health care delivery than the proposed out-of-hours ED and GPC should be considered. For inner-city hospitals with many self-referrals presenting during office hours, an integrated ED and GPC which functions 24 h a day, 7 days a week, might work. Other options are GPs working in the ED, or the combination of emergency nurse practitioners (ENPs) with emergency physicians. Non-urgent self-referrals can be handled by ENPs via a separate stream for minor injuries and minor illnesses. For more complex problems, an emergency physician or a GP is available, and for self-referrals with major trauma or needing specialist care, emergency physicians are available.

In order to allow for such alternative models of acute care delivery, tariffs for acute care should be independent of the provider of care. This enables efficient deployment of GPCs, independently contracted GPs, ENPs or other professionals tailored to the patient population and health care setting at hand.

We recommend further research into different models of care, their clinical outcomes and cost-effectiveness, and in ways to discriminate between patients needing hospital emergency care and patients who can be managed by the GP. Based on the findings in this study, ‘self-referral’ as an indicator for eligibility for GP care is not useful.

### Limitations

Firstly, because participating nurses only interviewed patients in their own shift, 42% of the self-referrals could not be asked for their motives for presenting to the ED. Nurses were instructed to include all self-referrals presenting during their shifts. However, participating nurses worked more often during out-of-hours, resulting in more interviewed self-referrals during out-of-hours than during office hours and in more non-injured self-referrals than injured self-referrals being interviewed. Subgroup analysis showed some differences in percentages of the motives given during office hours and out-of-hours; however, overall conclusions were not influenced and the order of frequency was unchanged. The oral interviewing allowed patients who would have been unable to fill out a questionnaire due to illiteracy to participate. With over 1,700 answers, it is unlikely that collecting additional interviews would have revealed other themes. Furthermore, we believe that both major motives (‘accessibility and convenience’ and ‘medical necessity’) would rather increase than decrease when more self-referrals presenting with injuries were asked for their motives. This suggests our results underestimate the importance of these motives.

Our criterion ‘needing hospital emergency care’ is not validated. Misclassification can go both ways: some patients classified as needing hospital emergency care might be equally well managed in primary care, and some patients might need hospital emergency care but did not fit in our criteria of needing hospital emergency care. An example of the first possibility is a patient with a headache, classified as ‘needing hospital emergency care’ based on having a CT scan performed to rule out brain haemorrhage. This patient was discharged home after excluding a brain haemorrhage. However, the GP might not have referred this patient to the ED for a diagnostic test to begin with. An example of the second possibility is a patient with a minor arterial bleeding, requiring surgical wound care. This patient is neither admitted nor suffering from an extremity fracture, does not need any diagnostic test and would thus be considered ‘not needing hospital emergency care’ in our definition.

We considered patients arriving by ambulance as non-self-referred, actually needing emergency care, because Dutch ambulance nurses are trained to assess whether a subject should be presented to the ED. It is therefore less likely that there were patients ‘suitable for GP care’ among the patients arriving by ambulance.

Finally, our study was conducted in a single inner-city institution; therefore, the results may not be generalizable to hospitals with a different ED patient case mix.

## Conclusions

In this inner-city hospital, 60% of the ED patients were self-referred. A substantial part of these patients needed hospital emergency care. Another part could have been treated by a GP, for many non-urgent as well as several urgent problems during office hours as well as out-of-hours.

We advocate for a health care system that is the same 24 h a day, 7 days a week, with one access point to medical care. Provision of acute care should be tailored to the patient population and health care setting. This includes efficient use of medical personnel (GPs, ENPs, emergency physicians, etc.) skilled to evaluate and/or treat the problem at hand, be it urgent, acute, complex, nocturnal or none of the above.

## Abbreviations

*B*: the coefficient for the constant

CI: confidence interval

CT: computerized tomography

ED: emergency department

EKG: electrocardiogram

ENP: emergency nurse practitioner

GP: general practitioner

GPC: general practitioner cooperative

METC: medical research ethics committee

MRI: magnetic resonance imaging

OR: odds ratio

PASW: Predictive Analytics Software

SD: standard deviation

SE: standard error around the coefficient for the constant

## Competing interests

The authors declare that they have no competing interests.

## Authors’ contributions

MCL had full access to all of the data in the study and takes responsibility for the integrity of the data and the accuracy of the data analysis. MCL, RL, CLB, NL and RCL contributed to the study concept and design. MCL acquired the data. MCL, RL, NL and RCL analysed and interpreted the data. MCL, RL, NL, CLB and CL drafted the manuscript. RL, CL, RH and JCG critically revised the manuscript for important intellectual content. All authors read and approved the final manuscript.
